# Is there an excess of significant findings in published studies of psychotherapy for depression?

**DOI:** 10.1017/S0033291714001421

**Published:** 2014-07-25

**Authors:** J. Flint, P. Cuijpers, J. Horder, S. L. Koole, M. R. Munafò

**Affiliations:** 1 Wellcome Trust Centre for Human Genetics, University of Oxford, UK; 2 Department of Clinical Psychology, VU University Amsterdam, The Netherlands; 3 Department of Forensic and Neurodevelopmental Sciences, Institute of Psychiatry, King's College London, UK; 4 UK Centre for Tobacco and Alcohol Studies, School of Experimental Psychology, University of Bristol, UK; 5 MRC Integrative Epidemiology Unit (IEU), at the University of Bristol, UK

**Keywords:** Cognitive behavioural therapy, depression, excess of significance, meta-analysis, psychotherapy, publication bias

## Abstract

**Background:**

Many studies have examined the efficacy of psychotherapy for major depressive disorder (MDD) but publication bias against null results may exist in this literature. However, to date, the presence of an excess of significant findings in this literature has not been explicitly tested.

**Method:**

We used a database of 1344 articles on the psychological treatment of depression, identified through systematic search in PubMed, PsycINFO, EMBASE and the Cochrane database of randomized trials. From these we identified 149 studies eligible for inclusion that provided 212 comparisons. We tested for an excess of significant findings using the method developed by Ioannidis and Trikalinos (2007), and compared the distribution of *p* values in this literature with the distribution in the antidepressant literature, where publication bias is known to be operating.

**Results:**

The average statistical power to detect the effect size indicated by the meta-analysis was 49%. A total of 123 comparisons (58%) reported a statistically significant difference between treatment and control groups, but on the basis of the average power observed, we would only have expected 104 (i.e. 49%) to do so. There was therefore evidence of an excess of significance in this literature (*p* = 0.010). Similar results were obtained when these analyses were restricted to studies including a cognitive behavioural therapy (CBT) arm. Finally, the distribution of *p* values for psychotherapy studies resembled that for published antidepressant studies, where publication bias against null results has already been established.

**Conclusions:**

The small average size of individual psychotherapy studies is only sufficient to detect large effects. Our results indicate an excess of significant findings relative to what would be expected, given the average statistical power of studies of psychotherapy for major depression.

## Introduction

Many studies have examined the efficacy of psychotherapy for major depressive disorder (MDD), and have established that psychotherapy is effective in the treatment of psychiatry's commonest illness (Elkin *et al.* 1989). Meta-analyses of these primary studies indicate that the effect of psychotherapies on MDD is comparable to those of antidepressant medications (Cuijpers *et al.* 2008*b*). However, there seems to be publication bias against null results for studies of both psychotherapies (Cuijpers *et al.* 2010, 2011) and antidepressant medications (Kirsch *et al.* 2008; Turner *et al.* 2008).

The existence of unpublished null findings means that the published literature contains an over-representation of positive findings and, as a result, corresponding estimates of effect size are likely to be inflated, overstating the efficacy of the intervention. Several factors may contribute to publication bias, including the reluctance of journals to publish null results and the lack of incentives for authors to invest time in writing up these studies (which are generally regarded as ‘less interesting’). In the case of studies of antidepressant medication, publication bias is also often attributed, at least in part, to the motivation of the pharmaceutical industry to suppress unfavourable results for commercial reasons. However, this motivation would not seem to apply to studies of psychotherapy.

There is growing evidence for the existence of publication bias in the psychotherapy literature, similar to that observed in the antidepressant literature. For example, Cuijpers *et al.* (2010) estimated from 89 studies of the efficacy of cognitive behavioural therapy (CBT) that the equivalent of 26 null studies remained unpublished, and statistically adjusting for this publication bias reduced the pooled effect size considerably. This adjustment is likely to be conservative because it depends on an analysis of a funnel plot, in which the study size (i.e. precision) is compared to the reported effect size. The idea underlying a funnel plot is that smaller studies are more likely to be published if they have larger than average effect sizes, resulting in an asymmetrical distribution around the pooled effect size. However, this method is relatively insensitive, particularly when there is a narrow range of sample sizes within the studies contributing to the meta-analysis (Lau *et al.* 2006). Funnel plots may therefore not be an effective diagnostic method for assessing the psychotherapy literature, where studies tend to be similar in size.

Tests of small-study effects are used to evaluate whether effect sizes are related to study size (e.g. funnel plot methods). An alternative approach is to test for an excess of statistically significant findings, which more directly evaluates whether the number of statistically significant results in a corpus of studies is higher than would be expected given a plausible estimate of the likely true effect size. This method, developed by Ioannidis & Trikalinos (2007), has been used previously to investigate ‘excess of significance’ in specific literatures (Ioannidis, 2011; Button *et al.* 2013*a*,*b*; Murphy *et al.* 2013). It typically uses meta-analysis of the literature to arrive at an estimate of the likely true population effect size and then, given the power of each individual study to detect an effect of that magnitude, compares the expected with the observed (i.e. published) number of significant findings.

We therefore set out to apply this test to studies of psychotherapy for depression, using an updated database of studies that has been used in a series of previous meta-analyses (Cuijpers *et al.* 2008*b*, 2011).

## Method

### Identification and selection of studies

**Fig. 1.**
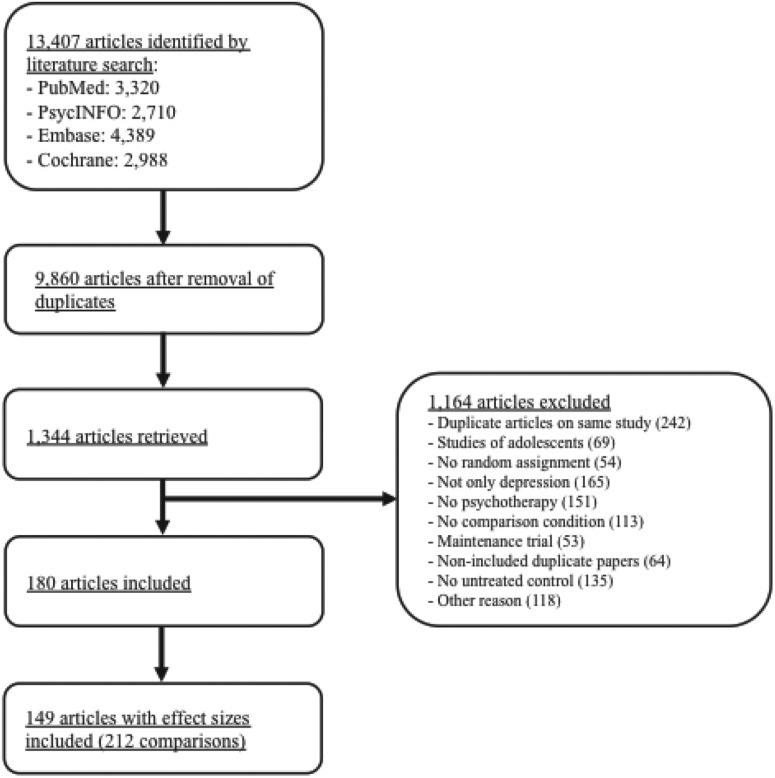
Flowchart of inclusion of studies.

We used a database of 1344 articles on the psychological treatment of depression that has been described in detail elsewhere (Cuijpers *et al.* 2008*b*, 2011), and that has been used in a series of earlier published meta-analyses (www.evidencebasedpsychotherapies.org). This database is continuously updated through comprehensive literature searches (currently from 1966 to January 2012). We examined 13 407 abstracts identified from PubMed (3320 abstracts), PsycINFO (2710), EMBASE (4389) and the Cochrane Central Register of Controlled Trials (2988). These abstracts were identified by combining terms indicative of psychological treatment and depression (both MeSH terms and text words). We also searched the primary studies from 42 meta-analyses of psychological treatment for depression to ensure that no published studies were missed. From the 13 407 abstracts, we identified 9860 unique abstracts after the removal of duplicates. Of these, 8516 were excluded based on the title and abstract, so that 1344 full-text articles were retrieved for possible inclusion in the database. Of these, 1164 articles were excluded (Fig. 1), resulting in the inclusion of 180 articles in the database.

For the present meta-analysis, included studies were randomized controlled trials in which a psychological intervention was compared to a control condition (waiting list; usual care; placebo; other) in people with depression (defined as an MDD according to a diagnostic interview, or as scoring above a cut-off on a self-report instrument). Excluded studies were studies of in-patients and adolescents (<18 years), and studies in which the effect size could not be calculated exactly (typically because only an overall *p* value was given for the comparison between treatment and control group at post-test, and no other information could be used to calculate the effect size). Co-morbid general medical or psychiatric disorders were not an exclusion criterion, and no language restriction was applied.

### Quality assessment

We assessed the validity of included studies using four criteria of the ‘risk of bias’ assessment tool, developed by the Cochrane Collaboration (Higgins & Green, 2011). This tool assesses possible sources of bias in randomized trials, including: the adequate generation of allocation sequence; the concealment of allocation to conditions; the prevention of knowledge of the allocated intervention (masking of assessors); and dealing with incomplete outcome data [this was assessed as positive when intent-to-treat (ITT) analyses were conducted, meaning that all randomized patients were included in the analyses]. The assessment tool includes two other criteria: evidence of selective outcome reporting; and other problems that could put it at a high risk of bias. The latter two criteria were not used in the present research because we found no indication in any of the studies that these had influenced the validity of the study.

### Statistical analysis

We first calculated individual study effect sizes, reflecting the difference between the psychotherapy group and the control group at post-test (Hedges' *g* or standardized mean difference). Effect sizes were calculated by subtracting (at post-test) the average score of the psychotherapy group from the average score of the control group, and dividing the result by the pooled standard deviations of the two groups. As several studies had relatively small sample sizes, we corrected the effect size for small sample bias according to the procedure suggested by Hedges & Olkin (1985), which corrects the pooled standard deviation to provide a more unbiased estimate of the population effect size. When calculating effect sizes, we only used those instruments that explicitly measured symptoms of depression, such as the Beck Depression Inventory (BDI) or the Hamilton Depression Rating Scale (HAMD). If more than one depression measure was used, the mean of the effect sizes was calculated, so that each comparison yielded only one effect size. If means and standard deviations were not reported, we calculated the effect size using dichotomous outcomes or other statistics that were available for calculating effect sizes (e.g. *t* statistic or *p* value).

We next calculated the summary effect size within both fixed and random effects frameworks. To estimate the heterogeneity of individual study effect sizes, we calculated the *I*^2^ statistic. A value of 0% indicates no observed heterogeneity, with larger values indicating increasing heterogeneity. Conventionally, 25% is regarded as low, 50% as moderate and 75% as high heterogeneity (Higgins *et al.* 2003). We also calculated the *Q* statistic. We explored the impact of study-level characteristics by stratifying our analysis by: analysis (ITT, per protocol); independent randomization (yes, no); control group (usual care, wait list, other); blinding of assessors (yes, no, not known); and country (UK, EU, USA, Australia, Canada, Other). We also conducted a meta-regression of effect size estimate on number of treatment sessions. Analyses were conducted using Comprehensive Meta-Analysis version 2.2.021 (Biostat, USA).

Finally, we calculated the achieved power for each study to detect the estimated summary effect reported in the meta-analysis, assuming an *α* level of 5%. Power was calculated using G*Power software (Faul *et al.* 2007). We then calculated the mean and median statistical power across all studies. The number of expected studies with statistically significant results was estimated, based on the average statistical power of individual studies given the likely true effect size, and compared against the number of observed significant studies to test for an excess of statistically significant results (Ioannidis & Trikalinos, 2007). This approach is conservative because it is based upon observed significant findings from those individual study effects in our meta-analysis. These effect sizes do not include adjustment for covariates as may have been the case in the published reports of those data.

## Results

### Description of studies

From the 180 articles included in the database, 149 were eligible for inclusion in our analysis. Of these, 49 included more than one active treatment arm, so that there were a total of 212 comparisons between psychotherapy and control conditions. The characteristics of the included studies are shown in the online Supplementary Table S1.

### Meta-analysis

Meta-analysis of the 149 psychotherapy studies identified by our search strategy indicated a summary effect size of *d* = 0.55 (95% CI 0.52–0.58, *p* < 0.001) within a fixed effects framework and *d* = 0.65 (95% CI 0.57–0.72, *p* < 0.001) within a random effects framework. There was evidence of substantial heterogeneity (*I*^2^ = 72%, *Q*_148_ = 625, *p* < 0.001).

The 92 studies that included a CBT arm indicated a summary effect size of *d* = 0.58 (95% CI 0.54–0.63, *p* < 0.001) within a fixed effects framework and *d* = 0.71 (95% CI 0.61–0.80, *p* < 0.001) within a random effects framework. There was again evidence of substantial heterogeneity (*I*^2^ = 79%, *Q*_91_ = 429, *p* < 0.001).

Stratification by study-level characteristics indicated that the summary effect size estimate was larger when analysis was per protocol rather than ITT, when there was no independent randomization, and when a wait list control was used. Meta-regression indicated a weak positive association between effect size and number of treatment sessions (slope 0.009, 95% CI 0.001–0.018, *p* = 0.032). These results are summarized in Table 1.

**Table 1.** Meta-analysis stratified by study-level characteristics

	*k*	*g*	95% CI	*p* value	*I*^2^ (%)	*p*_diff_
All studies						
Fixed effects	149	0.55	0.52–0.59	<0.001	76	n.a.
Random effects		0.65	0.57–0.72	<0.001		
CBT studies						
Fixed effects	92	0.58	0.54–0.63	<0.001	79	n.a.
Random effects		0.71	0.61–0.80	<0.001		
Analysis						
ITT	82	0.54	0.46–0.62	<0.001	71	0.002
Per protocol	67	0.80	0.65–0.96	<0.001	79	
Randomization^a^						
Yes	62	0.49	0.41–0.58	<0.001	70	<0.001
No	87	0.79	0.66–0.91	<0.001	77	
Control group						
Other	28	0.40	0.26–0.53	<0.001	68	<0.001
Usual care	61	0.55	0.45–0.66	<0.001	71	
Wait list	60	0.87	0.75–1.01	<0.001	77	
Blinding^b^						
Yes	122	0.61	0.54–0.68	<0.001	69	0.26
No	15	0.82	0.52–1.12	<0.001	72	
Not known	12	0.83	0.40–1.26	<0.001	93	
Country						
UK	22	0.43	0.31–0.55	<0.001	42	0.004
EU	24	0.53	0.41–0.66	<0.001	59	
USA	75	0.64	0.53–0.74	<0.001	71	
Australia	9	0.65	0.30–1.00	<0.001	79	
Canada	5	0.87	0.24–1.50	0.007	81	
Other	14	1.10	0.76–1.45	<0.001	92	

CBT, Cognitive behavioural therapy; ITT, intent to treat; CI, confidence interval; n.a., not applicable.

Stratified analyses were conducted within a random effects framework.

a Independent randomization.

b Blinding of assessors.

### Excess significance in all psychotherapy studies

For all 212 psychotherapy comparisons, the mean sample size was 35 (median 22) in the treatment group and 34 (median 21) in the control group. Assuming the summary effect size for all psychotherapy studies (*d* = 0.55) indicated by our meta-analysis represents a reasonable estimate of the true population effect size, we calculated the power of each comparison to detect such an effect. This indicated that the average statistical power was 49%. A total of 123 comparisons (58%) reported a statistically significant (i.e. *p* < 0.05) difference between treatment and control groups, However, on the basis of the average power observed, we would only expect 104 (i.e. 49%) to do so. There was therefore evidence of an excess of significance in this literature (*p* = 0.010). These results did not change substantively when the 149 individual studies (rather than comparisons) were considered (observed: 96, expected: 84, *p* = 0.051).

### Excess significance in CBT studies

We next restricted our analysis to the 139 comparisons that included a CBT arm. The mean sample size was 36 (median 23) in the treatment group and 36 (median 23) in the control group. The summary effect size for all CBT studies (*d* = 0.58) indicated that the average statistical power was 53%. A total of 87 comparisons (63%) reported a statistically significant difference between treatment and control groups whereas we would expect only 73 (i.e. 53%) to do so. Therefore, again, there was evidence of an excess of significance in this literature (*p* = 0.028). These results did not change substantively when the 92 individual studies (rather than comparisons) were considered (observed: 65, expected: 54, *p* = 0.006).

### p-value distributions for antidepressant and psychotherapy studies

We know empirically that publication bias operates in the antidepressant literature (i.e. some studies remained unpublished), from data obtained from the US Food and Drug Administration (Kirsch *et al.* 2008). It is therefore of interest to directly compare the antidepressant and psychotherapy literatures. One way of doing this is to compare the distribution of *p* values in both literatures. We used data from published and unpublished clinical trials of four antidepressants (Kirsch *et al.* 2008; Horder *et al.* 2011) to compare the distribution of *p* values for all studies, and for published studies only, within specific ranges (<0.01, 0.01–0.05, 0.05–0.10, >0.10).

**Fig. 2.**
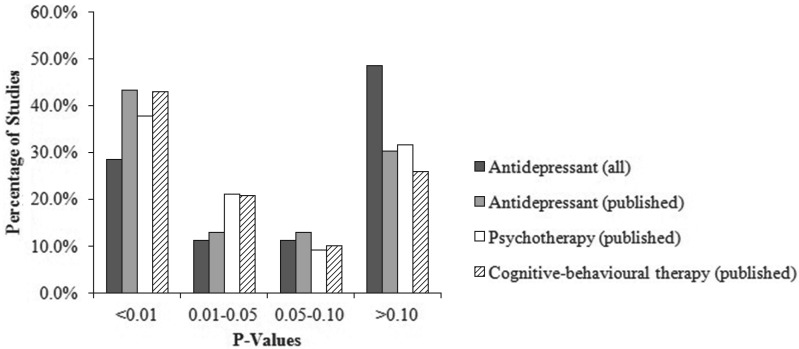
Proportion of published psychotherapy studies, and published and unpublished antidepressant studies, reporting *p* values within a specific range. The proportion of studies reporting *p* values within a specific range are shown for all antidepressant studies (*k* = 35), published antidepressant studies only (*k* = 23), published psychotherapy comparisons (*k* = 212), and published psychotherapy comparisons that included a cognitive behavioural (CBT) therapy arm (*k* = 139). In both the latter cases, the observed distributions resemble the distribution of published antidepressant studies only, where we know publication bias is operating.

The entire antidepressant literature contains several studies where *p* > 0.10, but this proportion is lower among published antidepressant studies, with a corresponding increase in the proportion of studies where *p* < 0.01. This is consistent with what we would expect to see if publication bias against null results is operating. We also plotted the distribution of *p* values for all psychotherapy studies, and only those studies that included a CBT arm. In both cases, the observed distributions resemble the published (i.e. biased) rather than total (i.e. published and unpublished) literature on antidepressant studies. These results are shown in Fig. 2.

## Discussion

Our analysis of studies of the effectiveness of psychotherapy for MDD indicates that there is an excess of significant findings relative to what would be expected given the average statistical power of these studies. These results were not altered when we restricted our analysis to studies of CBT. The distribution of *p* values in this literature also resembles that of the published antidepressant literature, where publication bias against null results has already been established. We also noted the small average size of individual psychotherapy studies, which would only be sufficient to detect relatively large effects. An excess of significance in a specific literature may be due to several factors, including null results remaining unpublished and null results that are presented as positive. The prevalence of unpublished null findings has previously been reported through meta-analysis of the psychotherapy literature (Cuijpers *et al.* 2010). Moreover, the misrepresentation of null findings has been documented in trials of antidepressant drugs (Turner *et al.* 2008). The latter bias seems to be operating also in the field of psychotherapy treatment, despite an apparent lack of overt financial incentives to do so. This is perhaps not surprising, given growing evidence that similar patterns are present across a diverse range of literatures and methodologies (Button *et al.* 2013*a*,*b*).

There are some limitations to our study to be considered. First, our meta-analysis indicated substantial between-study heterogeneity, making the choice of an appropriate effect size for the excess of significance test difficult. Simulations suggest that the most appropriate effect size to use when testing for excess significance is that derived from a fixed effects meta-analysis, or the effect size of the largest study included in each meta-analysis (Ioannidis, 2013). This is because effect sizes from random effects meta-analysis are typically larger than those from fixed effects meta-analysis (albeit with wider CIs), and they are particularly prone to inflation in the presence of reporting biases that predominantly affect smaller studies (Ioannidis, 2013).

Second, we did not include adjustment for covariates when calculating individual study effect sizes. However, given that all studies were randomized, we do not view this as a major limitation. Rather, our approach ensures that all data are treated in the same way, removing the scope for ‘researcher degrees of freedom’ (Simmons *et al.* 2011) influencing the effect size estimate for individual studies.

Third, we combined all psychotherapy approaches, and only conducted separate analyses for CBT. This was in part a pragmatic decision, given the large number of psychotherapy approaches. However, there seem to be few differences between the effects of different types of psychotherapies for depression (Cuijpers *et al.* 2008*a*; Barth *et al.* 2013). Nevertheless, it may be that the problem of excess significance is more pronounced for some literatures than others.

Fourth, our findings depend on the choice of a plausible effect size: the larger the effect, the larger the power of each study to detect that effect. The estimates from the meta-analysis are likely to be upwardly biased (for example, because of publication bias). Therefore, we suggest that our estimate of the excess of significance in this literature is likely to be conservative because, were the true effect size in fact less than the average published effect size, the average power of the studies to detect this effect would be even smaller.

Fifth, the true population effect size may vary systematically with sample size; for example, if there are cultural differences in the efficacy of psychotherapy for depression, the population effect size may vary across countries. If scientists within those countries are aware of this, they may calculate their sample sizes accordingly. Other methods have been developed to test for excess of significance, such as calculating the *post-hoc* power for individual studies, which could accommodate this. However, this method has typically been used when an effect size estimate from a meta-analysis is not available (Francis, 2012). Given that there are no strong reasons to believe that the true population effect size varies systematically with sample size, and that an effect size estimate derived from a meta-analysis is available, we consider our approach is most appropriate.

In conclusion, there exists an excess of significance in the literature on psychotherapy for depression. Although similar observations have been made of the antidepressant literature, it is instructive to see that an excess of significance can occur in a literature where financial vested interests are less likely to play a part. Our results have potentially important implications; in particular, the excess of significance we have observed in this literature, together with previous evidence of publication bias, emphasizes the importance of publishing null trial results. The AllTrials campaign (www.alltrials.net/) calls for all past and present clinical trials to be registered and their results reported, so that the efficacy and effectiveness of treatments can be properly assessed. If null results remain unpublished, then it follows that psychotherapy for depression (in this case) may be less effective than the published literature would suggest. There is interest in providing psychotherapy on a large scale in the UK (Clark, 2011) and elsewhere, which would require substantial financial investment. Consequently, it would be prudent to rigorously establish the effectiveness of the therapies that might be so provided, and the likely magnitude of their effect.

## Supplementary material

For supplementary material accompanying this paper, please visit http://dx.doi.org/10.1017/S0033291714001421.
